# Notes and News

**Published:** 1898-07-09

**Authors:** 


### NOTES AND NEWS.

(Collected and Communicated.)

The Maidstone typhoid epidemic has cost the ratepayers upwards of £17,500.

Mr J. Jackson Clarke will lecture on "Deformities Caused by Badly-shaped Boots," at the City Orthopædic Hospital, on Tuesday, July 12th, at half-past five p.m.

The Dundee Infirmary has received a donation of £5,000 from the family of the late John Sharp, jute manufacturer, of Dundee, being £1,000 each from the four sons and the daughter of the deceased.

An article by Miss Annesley Kenealy, entitled "The Master Craftsman of Earlswood," appearing in *Pearson's Magazine* for July, illustrates the marvellous technical skill which may exist in an individual noticeably deficient in brain power from infancy and a "life case" at Earlswood Asylum.

Under the will of the late Sir Robert Rawlinson, formerly chief engineer inspector of the Local Government Board, who died on May 31st, aged 88 years, leaving personal estate of the value of £82,838, a portion of the residuary estate is left to the trustees of St. Thomas's Hospital for the general purposes of the hospital. The amount which the charity will ultimately receive will apparently amount to about £35,000.

From the long list of delegates from public authorities and county councils deputed to attend the sixteenth congress of the Sanitary Institute, to be held in Birmingham from September 27th to October 1st, the meeting is expected to prove an eminent success. Sir Joseph Fayrer, Bart., is the president of the congress, and Dr. Alexander Hill, Master of Downing College, and Vice-Chancellor of Cambridge University—an eloquent sanitarian—is to deliver the popular lecture. Excursions of hygienic interest are arranged. The Lord Mayor has invited the meeting to a conversazione, while a garden party at the Botanical Gardens, Edgbaston, will form one of the social attractions. A health exhibition is also arranged, as well as a section on domestic hygiene, presided over by the Lady Mayoress.

We understand that the promoters of the Medical Graduates' College and Hospital have already received a considerable amount of financial support, and that they have been able to purchase the building in Chenies Street, which it is proposed to utilise for the purposes of the institution.

The Melbourne Medical Officer of Health, in his report, contrasts the death-rate of 13·6 per 1,000 for the city of Melbourne and 15 per 1,000 for the suburbs of the city with the higher rate of London, and considers his record for the year most satisfactory. The mortality from typhoid still remains high in Melbourne—much higher than in Great Britain generally.

The *Medical News*, of New York, says justly enough that after medical and surgical preparations have been made on such a large scale for the preventive and curative treatment of yellow fever, dysentery, and similar tropical ailments, it seems like a bit of grim jesting on the part of disease that it should manifest itself in an outbreak of measles such as has occurred at Camp Merritt, where new cases have cropped up at the rate of about twenty a day.

In regard to the news received this week as to the large number of men wounded in the actions before Santiago, it is worthy of note that one of the common diseases of Cuba is tetanus, and it will be a matter of much interest to observe how far the soldiers, treated, as we may suppose they will be, on the most approved antiseptic principles, escape this terrible malady. It cannot be said that we know very much as to the best means of preventing the infection of tetanus, when once implanted in a wound, from taking root; but, so far as we do know, all seems to point to the necessity of applying strong antiseptics as early as possible—much stronger antiseptics than some of the "aseptic" school are accustomed to apply to living tissues.

The Rev. Richard Wilson, of St. Augustine's, Stepney, is advocating an admirable scheme for the care of the many small children of hop-pickers during the approaching "hopping season." He says: "One thing I am certain is urgently needed and is practical, and that is a temporary cottage hospital for children. The lot of a sick child in a dry season is bad; in a wet season it beggars description. However bad the child is, the mother must go picking, if it is only to pay the fare home. The places where the 'hopper' lives can best be compared to a draughty sort of cow-house, and here the mother must leave her child alone all day on a bundle of straw, or else she must chance the weather and take it with her into the hop-field. Attention and nursing, in either case, are out of the question. In the village near where I am going hop-picking this year I have secured an empty cottage, which I propose to use as a children's hospital. I have an experienced nurse to take charge. But I want funds for the absolutely necessary furnishing, and for the maintenance of the hospital during the hopping season. I do not propose to do this on an extravagant scale. For instance, excellent beds for babies can be made up in washing baskets, but we shall be obliged to have at least four attendants, and the expenses must be considerable, and at present I have only £35 subscribed."

